# Randomized Clinical Trial of Azithromycin vs. Erythromycin for the Treatment of Chlamydia Cervicitis in Pregnancy

**DOI:** 10.1155/S1064744996000671

**Published:** 1996

**Authors:** M. S. Edwards, R. B. Newman, S. G. Carter, F. W. LeBoeuf, M. K. Menard, K. P. Rainwater

**Affiliations:** Division of Maternal-Fetal Medicine Department of Obstetrics and Gynecology Medical University of South Carolina 171 Ashley Avenue Charleston SC 29425-2233 USA

## Abstract

*Objective:* The purpose of this study was to prospectively test the null hypothesis that there is no difference in the clinical effectiveness of azithromycin and erythromycin for the treatment of chlamydia cervicitis in pregnancy.

*Methods:* All antepartum obstetrical patients underwent routine screening for chlamydia cervicitis using a DNA probe assay (Gen-Probe Pace, San Diego, CA). Women who tested positive for chlamydia cervicitis were prospectively randomized to receive either azithromycin 1 g orally at enrollment, or erythromycin 500 mg orally 4 times a day for 7 days. Sexual partners were referred to the county health department for evaluation and treatment. A test of cure was repeated in 2 weeks. Results were analyzed by chi-square analysis and Fisher's exact test when indicated.

*Results:* One hundred forty women tested positive for chlamydia cervicitis and agreed to randomization. There were 4 (6.2%) treatment failures in the azithromycin group and 18 (27.7%) in the erythromycin group (*P* = 0.005). Gastrointestinal side effects were reported by 42 (65.5%) of the women taking erythromycin, but only 12 (19.4%) of those taking azithromycin (*P* < 0.002). Gastrointestinal side effects and resultant noncompliance were significantly related to treatment failure with erythromycin.

*Conclusions:* The findings of this study support the conclusion that a single dose of azithromycin is a significantly more effective and better tolerated treatment regimen for chlamydia cervicitis in pregnancy than erythromycin which is currently recommended.


Infectious Diseases in Obstetrics and Gynecology 4:333–337 (1996)
© 1997 Wiley-Liss, Inc.

### Randomized Clinical Trial of Azithromycin vs. Erythromycin for the Treatment of Chlamydia Cervicitis in Pregnancy

**M.S. Edwards, R.B. Newman,* S.G. Carter, F.W. LeBoeuf, M.K. Menard, and K.P. Rainwater**

*Division of Maternal-Fetal Medicine, Department of Obstetrics and Gynecology, Medical University of South Carolina, Charleston, SC*

ABSTRACT

***Objective:*** The purpose of this study was to prospectively test the null hypothesis that there is no difference in the clinical effectiveness of azithromycin and erythromycin for the treatment of chlamydia cervicitis in pregnancy.

***Methods:*** All antepartum obstetrical patients underwent routine screening for chlamydia cervicitis using a DNA probe assay (Gen-Probe Pace, San Diego, CA). Women who tested positive for chlamydia cervicitis were prospectively randomized to receive either azithromycin 1 g orally at enrollment, or erythromycin 500 mg orally 4 times a day for 7 days. Sexual partners were referred to the county health department for evaluation and treatment. A test of cure was repeated in 2 weeks. Results were analyzed by chi-square analysis and Fisher's exact test when indicated.

***Results:*** One hundred forty women tested positive for chlamydia cervicitis and agreed to randomization. There were 4 (6.2%) treatment failures in the azithromycin group and 18 (27.7%) in the erythromycin group ($P = 0.005$). Gastrointestinal side effects were reported by 42 (65.5%) of the women taking erythromycin, but only 12 (19.4%) of those taking azithromycin ($P < 0.002$). Gastrointestinal side effects and resultant noncompliance were significantly related to treatment failure with erythromycin.

***Conclusions:*** The findings of this study support the conclusion that a single dose of azithromycin is a significantly more effective and better tolerated treatment regimen for chlamydia cervicitis in pregnancy than erythromycin which is currently recommended. Infect. Dis. Obstet. Gynecol. 4: 333–337, 1996. © 1997 Wiley-Liss, Inc.

KEY WORDS

treatment failure; compliance; sexually transmitted disease

Screening for *Chlamydia trachomatis* cervicitis is currently recommended at both the initial prenatal visit and again in the third trimester due to its high prevalence and potential for both maternal and neonatal infectious morbidity. Doxycycline or tetracycline are the treatments of choice for chlamydial infection in the nonpregnant woman, however, erythromycin is recommended by the Centers for Disease Control (CDC) during pregnancy.[1] Due to its frequent dosing scheduling (4 times a day for 7 days) and a high rate of gastrointestinal side effects, compliance with erythromycin presents a major problem in treating chlamydia during pregnancy. Azithromycin, an azilide antibiotic, has been shown to have therapeutic equivalency with doxycycline in the nonpregnant popu-

Contract grant sponsor: Pfizer Pharmaceuticals.

*Correspondence to: Dr. Roger B. Newman, Department of Obstetrics and Gynecology, Medical University of South Carolina, 171 Ashley Avenue, Charleston, SC 29425-2233.

Received 19 September 1996
Accepted 17 February 1997

lation.[2] Azithromycin has several potential advantages that affect compliance including a single-dose regimen and a much lower rate of side effects.

The purpose of this study was to determine the effectiveness of a single dose of azithromycin in comparison with the standard 7-day erythromycin treatment regimen for chlamydia cervicitis during pregnancy.

### SUBJECTS AND METHODS

The reported study is a prospective randomized trial of azithromycin vs. erythromycin for the treatment of chlamydia cervicitis in pregnancy. The research protocol was approved by the Institutional Review Board of the Medical University of South Carolina.

Routine screening for chlamydia cervicitis and gonorrhea was performed on all antepartum patients at their first prenatal visit and again in the third trimester (32–36 weeks gestation). All pregnant women over the age of 15 years who tested positive for *Chlamydia trachomatis* by DNA hybridization (Gen-Probe Pace, San Diego, CA) from a cervical swab specimen were eligible for enrollment. The only exclusion to enrollment was an allergy to or intolerance of either azithromycin or erythromycin.

Informed consent was obtained from all eligible patients who agreed to participate in this study. Patients were then randomized, using a preestablished random number table, to one of the two treatment regimens: enteric-coated erythromycin base 500 mg tablets, administered orally before meals 4 times each day for 7 days, or azithromycin 250 mg tablets, 4 tablets taken orally at enrollment. Patients were provided with the assigned antibiotics as part of the study design.

No attempt was made to blind the study. All sexual partners were referred to the appropriate county health department for treatment. Patients were scheduled to return for repeat cervical culture (test of cure) using the DNA hybridization technique 2 weeks following initiation of antibiotic therapy. Questionnaires regarding demographic data, obstetrical history, sexual history, history of sexually transmitted diseases (STDs), compliance with the treatment regimen, side effects, interval sexual intercourse, and whether or not the sexual partner received treatment were given to the patient both before and after treatment. These questionnaires were used to analyze contributing factors which might have influenced treatment outcome.

**TABLE 1. Demographics of the study patients with chlamydia cervicitis (N = 130)**

| Demographics | Azithromycin group (n = 65) | Erythromycin group (n = 65) | *P* |
|---|---|---|---|
| Age (years) | 21.6 | 22.1 | 0.63 |
| Black race (%) | 82.1 | 80.6 | 0.83 |
| Gravidity | 2.4 | 2.7 | 0.17 |
| Parity | 1.0 | 1.1 | 0.46 |
| Gestational age at enrollment (weeks) | 20.4 | 28.6 | 0.002 |

Results from the test of cure were compared between the two treatment groups using chi-square analysis and Fisher's exact test when indicated. The sample size of 65 patients in each treatment group was selected in order to detect a 15% difference in efficacy with a 95% confidence interval and a power of 80%. A cure rate of 80% was estimated for the group given erythromycin.[3–5]

### RESULTS

One hundred forty obstetrical patients were enrolled following a positive DNA hybridization test for chlamydia cervicitis at the Medical University of South Carolina between April 1993 and July 1994. Ten patients were lost to follow-up and a test of cure was not obtained: 7 patients in the erythromycin group and 3 in the azithromycin group. Sixty-two of the 65 patients in the azithromycin group completed their post-treatment questionnaires, while 64 of the 65 patients in the erythromycin group completed the same form.

The two treatment groups were similar with respect to age, race, gravidity, and parity (Table 1). Unexpectedly, a difference of 8.2 weeks in gestational age was found between the two treatment groups (erythromycin 28.6 weeks; azithromycin 20.4 weeks; $P = 0.002$).

Perinatal outcome was not different between the two groups. The mean gestational ages at birth were 38.8 ± 1.6 and 38.0 ± 2.0 weeks for the azithromycin and erythromycin groups, respectively. In the azithromycin group, there were 6 (9.2%) preterm deliveries (<37 weeks) of which 3 were due to preterm premature rupture of the membranes (PROM). In the erythromycin group, there were 8 (12.3%) preterm deliveries of which 5 were due to preterm PROM. There were no stillbirths, neona-

TABLE 2. Efficacy of treatment based on a follow-up DNA hybridization test 2 weeks after treatment and the completion of the follow-up patient questionnaire

| | Azithromycin (n = 62) | Erythromycin (n = 64) | P | Odds ratio[a] |
|---|---|---|---|---|
| Treatment failure | 4 (6.2%)[b] | 18 (27.7%)[b] | 0.005 | 0.17 (0.05–0.59) |
| Compliance | 62 (100%) | 38 (59.4%) | 0.001 | N/A |
| Side effects | 12 (19.4%) | 42 (65.6%) | 0.002 | 0.13 (0.05–0.30) |
| Partner treated | 37 (59.7%) | 33 (51.6%) | 0.60 | 1.4 (0.65–3.00) |
| Interval intercourse | 23 (37.1%) | 16 (25.0%) | 0.16 | 1.8 (0.79–4.18) |

[a]Ninety-five percent confidence intervals in parentheses.
[b]Treatment failure rates based on all 65 patients in each group who had the follow-up DNA hybridization test.

tal deaths, or serious anomalies in either group. One child in the azithromycin group had polydactyly and one in the erythromycin group had a small ventricular septal defect that has not required surgical repair.

Treatment failure was detected 2 weeks after the prescribed treatment regimen in 18 (27.7%) patients in the erythromycin group but only 4 (6.2%) patients in the azithromycin group ($P$ = 0.005) (Table 2). There was also a significant increase in the frequency of side effects reported by the erythromycin group and a significant decrease in the degree of compliance (Table 2). No significant differences were reported in the number of active sexual partners treated by the differing health departments or in the frequency of reported sexual intercourse prior to the test of cure between the two groups (Table 2).

Of the 62 women completing their post-treatment questionnaire in the azithromycin group, 12 (19.4%) reported side effects. Seven (11.3%) experienced nausea and 4 (6.5%) had emesis. One other patient reported pruritus. Side effects were reported by 42 (65.6%) of the 64 women completing their questionnaire after erythromycin treatment. Thirteen (20.3%) reported nausea, 23 (35.9%) emesis, 3 (4.7%) diarrhea, 1 (1.6%) cramping, and 1 each reported dizziness and pruritus.

Patients who had significant side effects with erythromycin were noncompliant 55% of the time compared to only 16.7% ($P$ = 0.002) for patients who did not report any major side effects. The impact of compliance on the cure rate was dramatic (Fig. 1). For those patients receiving erythromycin, the cure rate was much lower (51.9%) for those women who self-reported noncompliance compared to those who reported compliance (86.8%; $P$ < 0.01). When only those patients in the erythromycin group who reported good compliance are considered, successful treatment was not significantly different between the groups (erythromycin compliant 86.8%; azithromycin 93.8%).

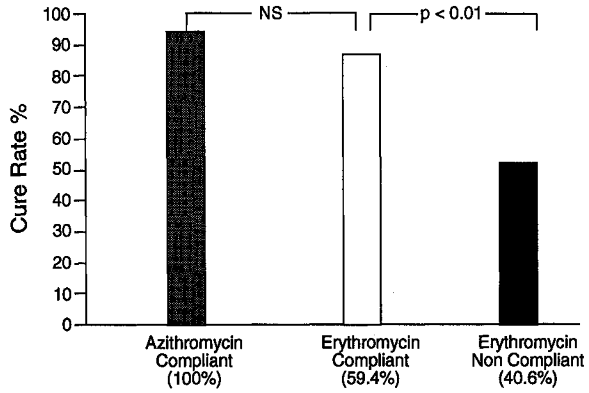

Fig. 1. Comparison of cure rates between the azithromycin group (N = 65) with observed 100% compliance and those patients receiving erythromycin who self-reported either compliance (N = 38) or noncompliance (N = 27) with the treatment regimen.

### DISCUSSION

*C. trachomatis* is a significant cause of both obstetrical and neonatal morbidity. Of those infants exposed to infected cervical secretions during delivery, up to 40% develop chlamydial conjunctivitis and 10–20% develop pneumonia.[6] *C. trachomatis* has also been associated with postpartum and postabortal endometritis.[5,7]

Erythromycin is the currently recommended antibiotic of choice for chlamydia cervicitis in pregnancy.[1] Erythromycin has been reported to have 76–100% efficacy in the treatment of chlamydial infections in nonpregnant populations.[3,4] An 83.3% cure rate was reported in a large, randomized treatment trial of chlamydia cervicitis in pregnancy.[5] However, in the current study, the recommended regimen of erythromycin base, 500 mg taken orally before meals 4 times daily, caused a significant number of patients to experience untoward gastrointestinal side effects, such as nausea, vomiting, gastritis, and diarrhea. These side effects, along with a difficult dosing regimen, contributed to a high rate of noncompliance with this antibiotic and a significantly higher failure rate.

A number of other antibiotic regimens have been studied in nonpregnant women for the treatment of chlamydia cervicitis, including tetracycline, doxycycline, sulfisoxazole, and several of the quinolones. However, these drugs are generally contraindicated in pregnancy. Clindamycin and amoxicillin have both been investigated during pregnancy and may be as efficacious as erythromycin in the treatment of chlamydia cervicitis.[5,8] While both of these antibiotics have fewer side effects than erythromycin, they also have a multidose daily treatment regimen that affects compliance.

Azithromycin is the prototype of a new class of antibiotics, the azilides. They have been shown to be as efficacious as doxycycline for the treatment of chlamydia cervicitis in nonpregnant women.[2] This was demonstrated with a single-stat 1 g dose of azithromycin compared to a standard 7-day doxycycline regimen of 100 mg twice a day. On reculture, 98% of women in both groups were free of disease.[2] In a single small investigation, the efficacy of azithromycin and erythromycin was compared in 30 pregnant women with cervical chlamydial infection.[9] Despite a high rate of gastrointestinal side effects in the subjects receiving erythromycin, there was no difference in efficacy between the two groups. Unfortunately, the small sample size prevented the investigation from having adequate power to discriminate between the two treatment regimens.

Azithromycin is closely related to erythromycin, although its pharmacokinetics differ greatly. Azithromycin differs from erythromycin due to an insertion of a methyl-substituted nitrogen atom into the aglycone ring. This insertion allows for rapid intracellular transfer which potentially makes it more effective against chlamydia, which is an obligate intracellular parasite. Erythromycin has less effective intracellular penetrance and therefore relies more heavily on eradication of the organism during the transient extracellular portion of chlamydia's 48–72-h life cycle. Because of these pharmacokinetics, erythromycin will only obtain comparable cure rates when it can achieve adequate serum levels for a more prolonged treatment course.

Although the available data are limited, there are no reported or anticipated differences in fetal risk between azithromycin and erythromycin. Both drugs carry a Pregnancy Category B designation with no known effects on pregnancy or the developing fetus.[10] It is noted that the current study has minimal power to detect any differences in perinatal outcome between the two drug regimens.

The results of this study demonstrate that a single-stat dose of azithromycin is more effective than a 7-day course of erythromycin for the treatment of chlamydia cervicitis in pregnancy. In fact, patients in the erythromycin treatment group had more than a four-fold increased risk of persistent chlamydia cervicitis at the 2-week follow-up testing compared to patients in the azithromycin group.

Treatment failures were seen in both antibiotic groups. Possible explanations include reinfection by an untreated partner, organism resistance to the drug, or incomplete penetration of the drug at the site of infection. It was anticipated that these factors would pose equivalent risks in both treatment groups. Indeed, no significant differences were seen in either the partner treatment rates or the reported rates of interim intercourse between the two groups.

Eligible patients were assigned to a treatment regimen based on a preestablished random number table as they were enrolled in the study without consideration of age, race, parity, or gestational age. No inherent bias can be identified in the study design. We cannot explain the difference in the mean gestational age between the two study groups except as an unlikely chance event. Although unlikely, it cannot be excluded that the difference in gestational age between the two groups could have affected the efficacy of one or the other treatment regimen. A greater propensity for gastrointestinal side effects might be expected at earlier gestational ages, but that was not the case in this study. The women receiving azithromycin did so at a significantly earlier gestational age, yet had fewer gastrointestinal side effects. Erythromycin, theoretically, could have been less effective at 28 weeks due to a larger blood volume of distribution compared to 20 weeks; however, the dosage of erythromycin used was standard for any point during pregnancy. Reinfection rates would also be expected to be lower at 28 weeks due to a reduced frequency of sexual intercourse with advancing gestation. However, while unlikely, it is not inconceivable that the difference in gestational age between the two study groups could have affected the outcomes of interest.

Treatment of chlamydia cervicitis with azithromycin is more expensive than treatment with erythromycin. However, the costs of retreatment and maternal and neonatal sequelae from persistent *C. trachomatis* infection are significant and help justify the additional expense of azithromycin. Based on its enhanced efficacy and compliance issues, we suggest that azithromycin be considered the treatment of choice for chlamydia cervicitis in pregnancy.

### ACKNOWLEDGMENTS

This study was supported in part by Pfizer Pharmaceuticals, which provided the azithromycin for this investigation.

### REFERENCES

1. Centers for Disease Control: 1989 Sexually transmitted diseases treatment guidelines. MMWR 38(Suppl 8):27–28, 1989.
2. Stamm WE: Azithromycin in the treatment of uncomplicated genital chlamydial infections. Am J Med 91(Suppl 3A):19S–22S, 1991.
3. Johannisson G, Lowhagen GB, Lyke E: Genital *Chlamydia trachomatis* infection in women. Obstet Gynecol 56:671–675, 1980.
4. Oriel JD, Ridgeway GL: Comparison of erythromycin and oxytetracycline in the treatment of cervical infection by *Chlamydia trachomatis*. J Infect 2:259–262, 1980.
5. Alger LS, Lovchik JC: Comparative efficacy of clindamycin versus erythromycin in eradication of antenatal *Chlamydia trachomatis*. Am J Obstet Gynecol 165:375–381, 1991.
6. Harrison HR, Alexander ER: Chlamydia Infections in Infants and Children. Sexually Transmitted Diseases. New York: McGraw-Hill, p 811, 1990.
7. Barbacci MB, Spence MR, Kappus EW, Burkman RC, Rao L, Quinn TC: Postabortal endometritis and isolation of *Chlamydia trachomatis*. Obstet Gynecol 68:686–690, 1986.
8. Crombleholme WR, Schachter J, Grossman M, Landers DV, Sweet RV: Amoxicillin therapy for *Chlamydia trachomatis* in pregnancy. Obstet Gynecol 75:752–756, 1990.
9. Bush MR, Rosa C: Azithromycin and erythromycin in the treatment of cervical chlamydial infection during pregnancy. Obstet Gynecol 84:61–63, 1994.
10. Hopkins S: Clinical toleration and safety of azithromycin. Am J Med 91(Suppl 3A):40S–45S, 1991.
